# Unloading Model of Elastic–Plastic Half-Space Contacted by an Elastic Spherical Indenter

**DOI:** 10.3390/ma17123018

**Published:** 2024-06-20

**Authors:** Wenhao Xie, Yuanyuan Guo, Huaiping Ding, Xiaochun Yin, Panpan Weng

**Affiliations:** 1 School of Physics, Nanjing University of Science and Technology, Nanjing 210094, China; wenhao_xie@njust.edu.cn (W.X.); guoyuanyuan@njust.edu.cn (Y.G.); ding_huaiping@njust.edu.cn (H.D.); yinxiaochun@njust.edu.cn (X.Y.); 2 School of Energy and Power Engineering, Nanjing University of Science and Technology, Nanjing 210094, China

**Keywords:** unloading, indentation contact, analysis model, residual deformation

## Abstract

A new unloading contact model of an elastic–perfectly plastic half-space indented by an elastic spherical indenter is presented analytically. The recovered deformation of the elastic indenter and the indented half-space has been found to be dependent on the elastic modulus ratio after fully unloading. The recovered deformation of the indented half-space can be calculated based on the deformation of the purely elastic indenter. The unloading process is assumed to be entirely elastic, and then the relationship of contact force and indentation can be determined based on the solved recovered deformation and conforms to Hertzian-type. The model can accurately predict the residual indentation and residual curvature radius after fully unloading. Numerical simulations are performed to demonstrate the assumptions and the unloading model. The proposed unloading model can cover a wide range of indentations and material properties and is compared with existing unloading models. The cyclic behavior including loading and unloading can be predicted by combining the proposed unloading law with the existing contact loading model. The combined model can be employed for low-velocity impact and nanoindentation tests and the comparison results are in good agreement.

## 1. Introduction

Contact is a fundamental problem and mainly studies the response of contact deformation and force. It is widely used in many subject areas and engineering applications, such as metal hardness testing [1], nanoindentation technology [2,3], thermal spraying [4], powder compaction [5] and gear meshing [6,7]. The frontier mechanical problem has important theoretical and engineering value. Unloading is also important in the elastic–plastic contact problem. The unloading law is commonly used to estimate energy loss [8,9], solve the coefficient of restitution [10,11] and measure material mechanical parameters [12,13,14]. An unloading model can quickly solve the contact response of force and displacement during the unloading process and calculate the residual deformation after unloading. The indentation unloading curve is the basis for the measurement of contact hardness and material properties [15,16,17].

At the beginning of unloading, the contact pressure, contact force and contact deformation reach their maximum value, which are the initial values of the unloading process. When the contact is elastic, the unloading behavior completely follows the Hertzian contact theory and the contact deformation can be completely recovered. When the contact is in an elastic–plastic deformation regime, permanent deformation and residual stress will occur after fully unloading [18]. The contour of the residual surface can be approximated as a sphere with a curvature radius $R_{res}$ [19]. Yan and Li [20] concluded that the unloading process is purely elastic and can be assumed as a reverse of reloading. However, plastic deformation could occur for some unloading processes [21]. It is noted that the effect of such unloading plastic deformation is small; the unloading behavior can still be considered to be purely elastic [22,23,24]. The existing elastic–plastic unloading models can be classified into two categories [25]: the Hertzian unloading model and the non-Hertzian unloading model.

For Hertzian-type unloading, Johnson [26] assumed the unloading law can be represented by the formula

(1) $$F=4E^{*}{R^{*e}}^{0.5}{\left(\delta -\delta_{r}\right)}^{1.5}/3,$$

where $R^{*e}$ and $\delta_{r}$ are the deformed effective curvature radius and residual indentation after unloading, respectively. Therefore, the unloading law can be determined by either $R^{*e}$ or $\delta_{r}$, which are dependent on the maximum contact force $F_{m}$ and indentation $\delta_{m}$. $R^{*e}$ can be expressed by the curvature radius of the spherical indenter and the residual contour. Stronge [27] obtained the relationship of the effective curvature radius $R^{*}$ in the loading regime and $R^{*e}$ in the unloading regime by geometry similarity,

(2) $${f_{u}\delta }_{Y}/R^{*}=\left(\delta_{m}-\delta_{r}\right)/R^{*e},$$

where $f_{u}=1$ for Stronge’s assumptions. Dong et al. [22] and Chen et al. [25] proposed a varying coefficient $f_{u}$ through the finite element (FE) results and impact simulations, which is dependent on the materials and maximum indentation. Thornton [28] assumed the unloading is elastic and the unrecoverable plastic deformation causes the contact geometry to flatten and the contact curvature to decrease. Then, he proposed a method to solve the equivalent curvature radius and found the recovery coefficient predicted by Stronge is larger at low speed. Vu-Quoc et al. [29] concluded the effective radius changes with the increasing contact deformation. Brake [30,31] concludes that the effective curvature radius varies from different loading stages. When unloaded at the full plastic deformation stage, $R^{*e}$ remains constant and can be corrected by geometric relations. Ma and Liu [32] provided the expressions of $R^{*e}$ at different loading stages according to the maximum contact force, critical yield contact force and critical full plastic contact force. Rathbone et al. [33] thought the unloading process obeys Hertzian-type and proposed the expression of $R^{*e}$ through linear fitting while considering the influence of Poisson’s ratio. The Hertzian-type unloading model can also be solved by $\delta_{r}$. Kogut and Komvopoulos [34] considered the unloading process obeys Hertzian theory and proposed a new formulation for permanent deformation based on FEM results. Jackson and Green studied the residual stresses and permanent deformation through FE results [18] and then proposed an empirical formula for the residual deformation [35]. Du and Wang [36] derived the expression of $\delta_{r}$ using a theoretical loading model based on the condition that the contact force at the end of loading is equal to that at the beginning of unloading. Ghaednia et al. [37] revised the coefficient of the formula and presented a new formula for the residual deformation by fitting from the impact experiment.

The unloading law of non-Hertzian-type does not follow the Hertzian force–indentation relation and has different unloading formulas. The unloading laws are usually determined from the FE simulations [38], experiments [39] and theoretical analysis [40]. Etsion et al. [24] concluded that the residual contour on the unloaded surface is not uniform and provided a new empirical expression for the unloading process based on FE results. Zhao et al. [41] followed Etsion et al.’s work and proposed analytical dimensionless expressions of the contact load, contact area and residual interference after fully unloading for the power-law hardening materials in the elastic–plastic case for a wide range of interferences. Majeed et al. [40] provided a simple contact model based on elastic–plastic and fully plastic indentation theory, which can be applied to low-velocity impact [42] for different structures, half-space or composite layers. Edmans and Sinka [43] provided insight into contact behavior during unloading by conducting a finite element study to characterize complex nonlinear features during unloading. They concluded that a single synthetic measure of initial particle deformation relative to deformation at first yield is insufficient to characterize the unloading response at large displacements when considering both stiffness and nonlinearity results. Ghaednia et al. presented new formulations for single asperity frictionless indentation contact [44] and flattening contact [45] considering the effect of bilinear strain hardening. The deformations on both of the objects, real contact radius and contact force have been considered in the model. They concluded that even 1% strain hardening causes significant changes in the contact parameters and contact profile. Chen et al. [19] investigated the unloading behavior of the elastic-power-law strain hardening half-space frictionlessly indented by the elastic sphere for systematic materials and they found the unloading curve follows a power-law relationship, whose exponent is sensitive to strain hardening but independent upon indenter elastic deformation. Then, an innovative contact unloading law of strain hardening materials is presented based on the power-law relationship of the unloading curve and the expression of the residual indentation fitted from the FE data.

Till now, studies on elastoplastic contact behavior and corresponding theoretical unloading models have mostly focused on the case of elastoplastic materials in contact with a rigid sphere [32,46,47] or rigid flat [33]. However, none of the objects can be considered rigid except symmetric contact. For those elastic–plastic contact laws originally derived by the conventional theoretical models, the effective elastic modulus $E^{*}$ is normally employed to account for the indenter elastic deformation, which causes significant deviations [23]. Therefore, the contact between an elastic indenter and an elastic–plastic half-space is considered to study and the elastic deformation of the indenter cannot be ignored. Especially in the nanoindentation measurement test, ignoring the elastic deformation of the indenter or using inaccurate expressions can result in obvious measurement errors [48].

This paper aims to obtain high-accuracy predictions of the indentation–force relationship of unloading, and a theoretical unloading model between the elastic sphere and elastic–plastic half-space is presented. The unloading model is derived based on the assumption of Hertzian theory and depends on the elastic deformation of the indenter. Numerical simulations have been performed to demonstrate the model can cover a wide range of material properties. Finally, the unloading model combined with the existing loading contact model can be employed to predict the response of the contact force and indentation.

## 2. Analysis Method

### 2.1. Hertzian Theory and Critical Indentation

**Figure 1:**
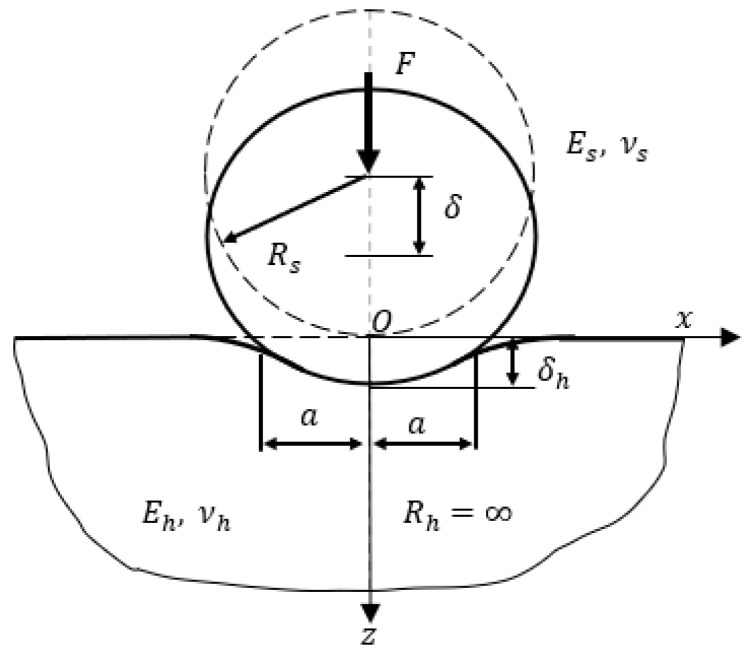
Schematic of elastic contact between half-space and sphere.

A typical frictionless and elastic contact of a sphere and a half-space is shown in Figure 1; $E_{s}$, $E_{h}$, $v_{s}$, $v_{f}$, $R_{s}$ and $R_{h}$ are the elastic modulus, Poisson’s ratio and the curvature radius of the elastic sphere and half-space, respectively.

The center displacement of the elastic–plastic half-space is $\delta_{h}$ and the center displacement of the elastic sphere is $\delta_{s}$, with the total displacement $\delta =\delta_{h}+\delta_{s}$. The analytical solution of the above elastic contact can be solved by Hertzian theory [49].

The pressure distribution between two frictionless elastic solids of revolution is given by Hertzian theory as

(3) $$p(r)=p_{0}{\left(a^{2}-r^{2}\right)}^{0.5}/a,$$

where $p_{0}$ is the maximum center pressure, r is the radial distance and a is the contact radius. The total load $F={2p_{0}\pi a}^{2}/3$ is solved by the integral of the elliptic pressure. The pressure distribution produces normal displacements $u_{z}$ of the elastic sphere and half-space, described by

(4) $$\begin{array}{cc} u_{zs}=\frac{1}{E_{s}^{*}}\frac{\pi p_{0}}{4a}(2a^{2}-r^{2}), & r\leq a \end{array},$$

(5) $$\begin{array}{cc} u_{zh}=\frac{1}{E_{h}^{*}}\frac{\pi p_{0}}{4a}(2a^{2}-r^{2}), & r\leq a \end{array}.$$

The equivalent Young’s modulus of half-space $E_{h}^{*}$ and sphere $E_{s}^{*}$ can be expressed as

(6) $$\begin{array}{cc} E_{h}^{*}=E_{h}/(1-\nu_{h}^{2}), & E_{s}^{*}=E_{s}/(1-\nu_{s}^{2}) \end{array}.$$

When r=0, $\delta_{s}$ and $\delta_{h}$ represent the displacements of the elastic sphere and half-space and $u_{zs}$, $u_{zh}$ can be expressed as

(7) $$\delta_{s}=u_{zs}=\pi p_{0}a/2E_{s}^{*}\begin{array}{cc} , & {\delta_{h}=u}_{zh}=\pi p_{0}a/2E_{h}^{*}. \end{array}$$

Based on the boundary conditions, the relationship of displacements can be obtained as

(8) $$u_{zs}{+u}_{zh}=\delta -(1/2R^{*})r^{2},$$

where the equivalent curvature radius during loading $R^{*}$ is expressed as

(9) $$1/R^{*}=1/R_{h}+1/R_{s}.$$

Substituting the expressions of $u_{zs}$ and $u_{zh}$ into Equation (8), one obtains

(10) $$\frac{\pi p_{0}}{4aE^{*}}(2a^{2}-r^{2})=\delta -(1/2R^{*})r^{2},$$

where the total equivalent Young’s modulus $E^{*}$ is expressed as

(11) $$1/E^{*}=1/E_{s}^{*}+1/E_{h}^{*}.$$

The contact radius a, the total indentation δ and the contact force F are given by

(12) $$a=\frac{{\pi p}_{0}R^{*}}{2E^{*}}={\left(\frac{3FR^{*}}{4E^{*}}\right)}^{1/3},$$

(13) $$\delta ={\left(a^{2}/R^{*}\right)}^{0.5},$$

(14) $$F=4E^{*}\sqrt{R^{*}}\delta^{1.5}/3.$$

According to the analysis (Equations (7)–(10)), the displacements of δ, $\delta_{s}$ and $\delta_{h}$ have a relation in the elastic regime,

(15) $$\begin{array}{ccc} E_{f}^{*}\delta_{f}=E_{s}^{*}\delta_{s}, & E_{f}^{*}\delta_{f}=E^{*}\delta , & E_{s}^{*}\delta_{s}=E^{*}\delta . \end{array}$$

For elastic–plastic contact, the contact plasticity will be generated as the contact force or indentation increases. The plastic yielding can be determined by the von Mises yield criterion,

(16) $$\sigma_{ep}=\sqrt{\frac{3}{2}{s_{ij}s}_{ij}}=\sigma_{Y},$$

where $\sigma_{eq}$ is the von Mises equivalent stress, and $\sigma_{Y}$ is the von Mises yield stress. According to the research [26], the yield criterion can be expressed as

(17) $$\begin{array}{ccc} \delta_{Y}={\left(\frac{{\pi C}_{\nu }\sigma_{Y}}{2E^{*}}\right)}^{2}R^{*}, & F_{Y}=\frac{{\left({\pi C}_{\nu }\sigma_{Y}\right)}^{3}{R^{*}}^{2}}{6{E^{*}}^{2}}, & a_{Y}=\frac{{\pi C}_{\nu }\sigma_{Y}R^{*}}{2E^{*}} \end{array},$$

where $\delta_{Y}$, $F_{Y}$ and $a_{Y}$ are the critical indentation, force and contact radius, respectively. $C_{\nu }$ is a function of Poisson’s ratio and the center contact pressure $p_{0}=C_{\nu }\sigma_{Y}$. $C_{\nu }$ has been solved numerically by Green [50] and then the numerical results were precisely fitted,

(18) $$C_{\nu }=1.30075+0.87825\nu +{0.54373\nu }^{2}.$$

For the special Poisson’s ratios ν=0.3, $C_{\nu }$ is equal to 1.61316.

### 2.2. Unloading Analysis

**Figure 2:**
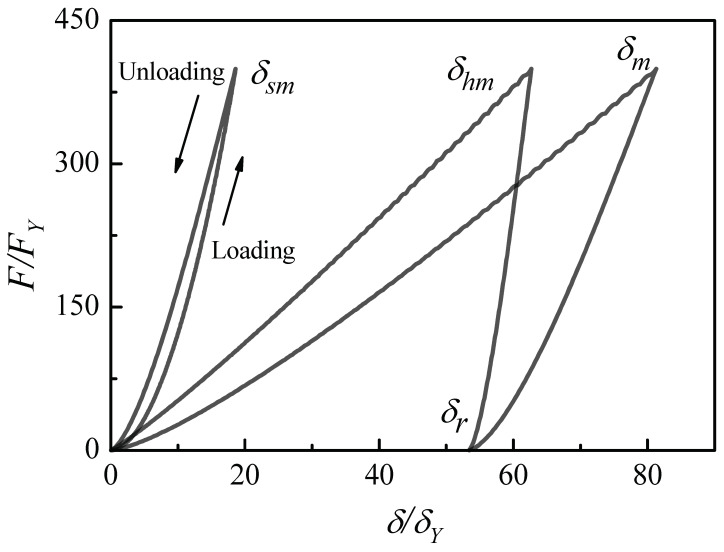
Loading and unloading contact between elastic–plastic half-space and elastic sphere.

For elastic–plastic contact, the loading and unloading curves of the indentation $\delta {/\delta }_{Y}$, ${\delta_{s}/\delta }_{Y}$ and ${\delta_{h}/\delta }_{Y}$ and contact force ${F/F}_{Y}$ are plotted in Figure 2.

At the end of the loading process, the total indentation δ unloads from the maximum indentation $\delta_{m}$ to residual indentation $\delta_{r}$, and the center displacement of the elastic–plastic half-space $\delta_{h}$ unloads from the maximum indentation $\delta_{hm}$ to residual indentation $\delta_{r}$. Due to it being fully elastic, the center displacement of the elastic sphere $\delta_{s}$ unloads from the maximum indentation $\delta_{sm}$ to residual indentation 0. It is noted that the $\delta_{s}$ curve on the right is loading and on the left is unloading, which is opposite to the total indentation δ and the center displacement of the half-space $\delta_{h}$.

According to the literature [20], the unloading constitutive equation can be considered to be purely elastic and follow the Hertzian solution,

(19) $$F=4E^{*}\sqrt{R^{*e}}{\left(\delta -\delta_{r}\right)}^{1.5}/3,$$

where $R^{*e}$ is the curvature radius of unloading. To solve the formula, the unloading process can be equivalent to a reversible loading phase [20] and can be solved by Hertzian loading theory [49]. The relationship of the total unloading deformation ($\delta_{m}-\delta_{r}$), the recovered displacement of the half-space ($\delta_{hm}-\delta_{r}$) and the sphere ($\delta_{sm}-0$) can be calculated by Equation (15) as follows,

(20) $$E_{s}^{*}\left(\delta_{sm}-0\right)=E^{*}\left(\delta_{m}-\delta_{r}\right),$$

(21) $$E_{h}^{*}\left(\delta_{hm}-\delta_{r}\right)=E^{*}\left(\delta_{m}-\delta_{r}\right),$$

(22) $$E_{h}^{*}\left(\delta_{hm}-\delta_{r}\right)=E_{s}^{*}\left(\delta_{sm}-0\right).$$

Then, the residual indentation $\delta_{r}$ can be solved by Equation (20),

(23) $$\delta_{r}=\delta_{m}-E_{s}^{*}\delta_{sm}/E^{*}.$$

Substituting Equation (23) into Equation (19), the unloading curvature radius $R^{*e}$ can be obtained at the maximum force $F_{m}$,

(24) $$R^{*e}={\left(3F_{m}/4{/E}^{*}\right)}^{2}/{\left(E_{s}^{*}\delta_{sm}/E^{*}\right)}^{3}.$$

The loading phase ends at a maximum load $F_{m}$ and generates the maximum deformation $\delta_{m}$, $\delta_{hm}$ and $\delta_{sm}$. Once the displacement $\delta_{sm}$ is known, the unloading law of Equation (19) can be obtained by Equations (23) and (24).

## 3. Validations

### 3.1. Finite Element Model

**Figure 3:**
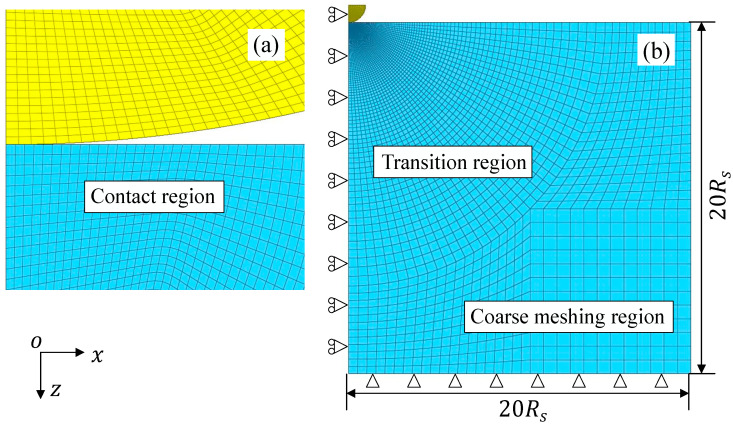
The mesh of the fundamental FE model: (**a**) contact region, (**b**) overall view of the mesh.

A frictionless contact of an elastic hemisphere with an elastic–perfectly plastic half-space is simulated to demonstrate the unloading theory using the FE software ANSYS 16.2 as shown in Figure 3. A hemisphere with radius $R_{s}=35\;mm$ is used to simulate an elastic sphere, and a cylinder with height and weight $20R_{s}$ is used to simulate an elastic–plastic half-space. The hemisphere is set to be fully elastic and the material of the half-space is elastic–perfectly plastic. The size convergence of the half-space is also satisfied, which is large enough that the boundary constraints can only have a little influence on the simulation results.

A typical isoperimetric rectangle axisymmetric 2-D element PLANE182 with four nodes is used to improve the efficiency of computation. In Figure 3a, the dense and regular elements are meshed around the contact area to obtain accurate results. In other regions, the element size is gradually changed for high computational efficiency, as can be seen in Figure 3b. For the contact conditions, Targe169 and Contact 171 elements are employed and the elements in the contact region are more than 50 to obtain an accurate contact radius. To deal with the high nonlinearity of the contact and finite plastic deformation, an iterative scheme of the full Newton–Raphson method is used to enhance the accuracy of the convergence solution [51]. The convergence criterion is controlled by the contact force. The convergence tolerance is 0.001 and the Euclidean norm (L2 norm) is used. For the boundary conditions, the boundary of the half-space is restricted in movement and the axis of symmetry can move vertically [52]. The hemisphere is loaded against the half-space by displacement control.

For fast modeling, the sub-models are created automatically by a program of the APDL language based on the fundamental FE model as shown in Figure 3. For different FE models with various maximum indentations, the half-space consists of 8914~12,914 elements with a total of 9161~13,261 nodes, and the sphere consists of 6920~10,800 elements with a total of 7124~11,101 nodes [23]. The contact radius can be obtained by the normal stress [29], the last node in contact [53] and the intersection point of two contact curves on the sphere and half-space [23]. Smooth results related to contact radius a can be obtained by the intersection point method [23]. More details of the FE model are introduced in the literature [23].

**Figure 4:**
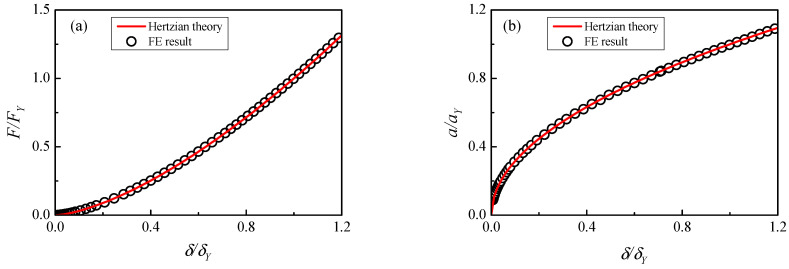
Comparison of FE results with Hertzian theory: (**a**) contact force, (**b**) contact radius.

To verify the accuracy of the above FE model, a purely elastic contact simulation is performed in Figure 4 and compared with the Hertz theory. It indicates that the FE solutions are in good coincidence with the theoretical solutions for contact radius and contact force. The error of the FE contact force F to the Hertzian solution is less than 0.1% and the error of the FE contact radius a to the Hertzian solution is less than 0.4%. To obtain higher precision in small indentations, the smaller contact area can be re-meshed to obtain the required computational accuracy.

The materials of the elastic–perfectly plastic half-space and elastic sphere are plotted in Table 1. Five indentation contact cases with different elastic modulus ratios are employed to simulate typical metals and alloys. Each material includes several FE sub-models with different contact areas and can cover a wide range of indentations, where the representative contact strain evolves from the elastic regime to ${a/R}^{*}=0.5$. The Poisson’s ratios $\upsilon_{s}=\upsilon_{h}=0.3$ and the elastic modulus ratio ${E_{h}^{*}/E}_{s}^{*}$ varies from 0.001 to 4. When the ${E_{h}^{*}/E}_{s}^{*}$ is small enough, the harder indenter can be assumed as a rigid indenter.

### 3.2. Hertzian Unloading

**Figure 5:**
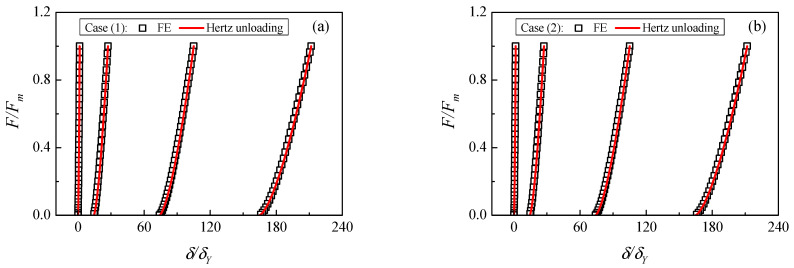
Validations of Hertzian unloading law: (**a**) material case (1), (**b**) material case (2).

Normally, the unloading process after elastic–plastic loading is assumed to be purely elastic and obeys the Hertzian relation according to the research [22,23,24]. Figure 5 shows the comparison of the Hertzian unloading law and FE data. The unloading curves are obtained from different maximum indentations with material case (1) and case (2). It is shown that the Hertzian unloading law is in good coincidence with the FE data. It illustrates that the Hertzian unloading law can accurately predict the evolutions of the contact force and indentation during the unloading process.

### 3.3. Residual Indentation

**Figure 6:**
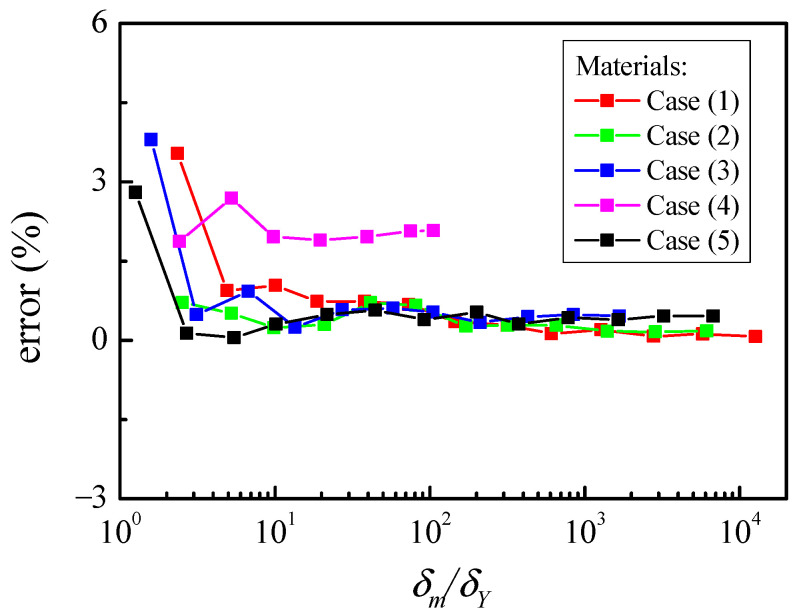
Error of the residual indentation $\delta_{r}$.

After fully unloading from elastic–plastic contact, the permanent deformation will exist and part of the energy is converted to plastic deformation. It is significant to calculate the residual indentation $\delta_{r}$ for unloading and reloading. Figure 6 shows the errors of the residual indentation $\delta_{r}$ between the FE results and the solutions (Equation (23)) for different maximum indentations $\delta_{m}/\delta_{Y}$ with the five materials in Table 1.

The solutions of residual indentation $\delta_{r}$ are calculated by the maximum force $F_{m}$ and indentation $\delta_{m}$, $\delta_{sm}$ from the FE data at the end of the loading process. Figure 6 shows the error decreases with the increasing $\delta_{m}/\delta_{Y}$. The elastic modulus ratio ${E_{h}}^{*}/{E_{s}}^{*}$ varies from 4 to 0.25 to consider the effects of the indenter elastic deformation for cases (1–4). ${E_{h}}^{*}/{E_{s}}^{*}=0.001$ of material case (5) is the limit case; the ${E_{s}}^{*}$ of the hemisphere is large enough and can be considered as a rigid hemisphere. In the smaller deformation where the contact is predominantly elastic, the residual indentation $\delta_{r}$ is very small and can cause large deviations. For large deformation, the relative error is less. When $\delta_{m}/\delta_{Y}>3$, the relative error is lower than 3 percent. When $\delta_{m}/\delta_{Y}>6$, the relative error is lower than 2 percent. For case (4), the elastic deformation is much bigger and the relative error is higher than the others. Therefore, the formula in Equation (23) can be fulfilled to obtain the accurate residual indentation $\delta_{r}$.

### 3.4. Residual Curvature Radius

**Figure 7:**
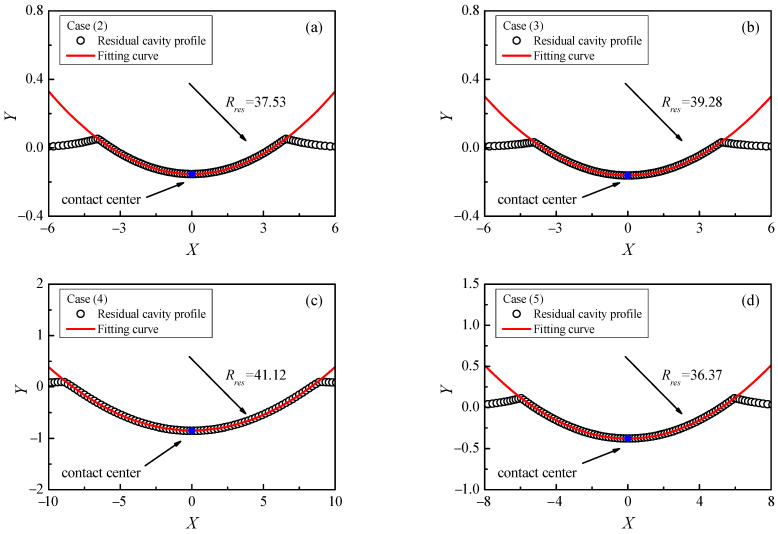
Comparison of residual curvature radius between Equation (25) and FE fitting: (**a**) material case (2), (**b**) material case (3), (**c**) material case (4), (**d**) material case (5).

After fully unloading from elastic–plastic contact deformation, the permanent deformation occurs on the contact surface of the half-space. The surface of the residual contour can be approximated as a sphere with a curvature radius $R_{res}$. Figure 7 shows the unloading residual deformation profile for four kinds of indentation contact cases in Table 1.

By spherical fitting, the curvature radius $R_{res}$ of the residual deformation profile is obtained. It can be seen that the spherical fitting is suitable for describing the residual deformation contour.

Table 2 shows the error of curvature radius $R_{res}$ between the fitting results and theoretical solution, which can be solved by the following,

(25) $$\frac{1}{R_{res}}=\frac{1}{R_{s}}-\frac{1}{R^{*e}},$$

where $R_{s}$ is the indenter radius and $R^{*e}$ is solved by Equation (24). The error is small and the maximum error is 1.39%. It indicates the effective curvature radius $R^{*e}$ determined by Equation (24) is suitable for obtaining the residual curvature radius $R_{res}$ after fully unloading.

### 3.5. Unloading Model

**Figure 8:**
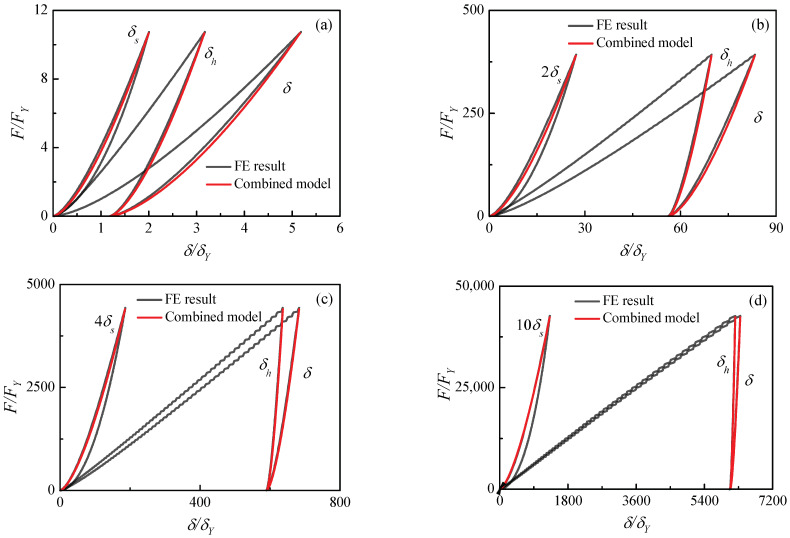
Comparisons of the unloading laws and FE results for different maximum indentations with material case (2): (**a**) 5.2$\delta_{Y}$ (**b**) 83.4$\delta_{Y}$, (**c**) 683.4$\delta_{Y}$, (**d**) 6346.9$\delta_{Y}$.

The unloading law (Equation (19)) can be validated by FE results. The unloading contrast of the contact force ${F/F}_{Y}$ and the contact indentation $\delta {/\delta }_{Y}$, ${\delta_{s}/\delta }_{Y}$ and ${\delta_{h}/\delta }_{Y}$ are plotted in Figure 8 for the elastic and elastic–plastic material case (2) in Table 1. Four unloading curves from different maximum indentations $\delta_{m}/\delta_{Y}=5.2$, $\delta_{m}/\delta_{Y}=83.4$, $\delta_{m}/\delta_{Y}=683.4$ and $\delta_{m}/\delta_{Y}=6346.9$ are obtained and shown in Figure 8a–d, respectively. It is noted that the loading curve is on the right and the unloading curve is on the left for the elastic hemisphere.

Figure 8a shows the small deformation where the contact is predominantly elastic, and the displacement of the elastic–plastic deformation $\delta_{h}$ and the elastic hemisphere $\delta_{s}$ are in the same magnitude.

The unloading law agrees well with the FE result and the residual indentation $\delta_{r}$ also shows excellent agreement with the FE result. Figure 8b shows a medium deformation where the plastic deformation is in dominant status, and the elastic hemisphere deformation is small. For a more clearer display, the displacement of the elastic hemisphere $\delta_{s}$ is magnified two times. The unloading model also compares well with the FE results. Figure 8c shows the large indentation where the plastic flow can move in a wider range. For a more clearer display, the displacement of the elastic hemisphere $\delta_{s}$ is magnified four times. Figure 8d shows a very large indentation where the pile-up formation of the contact area can be generated. For a more clearer display, the displacement of the elastic hemisphere $\delta_{s}$ is magnified 10 times. The contrast in Figure 8c,d illustrates that the unloading theory can be applied well to heavy indentations.

**Figure 9:**
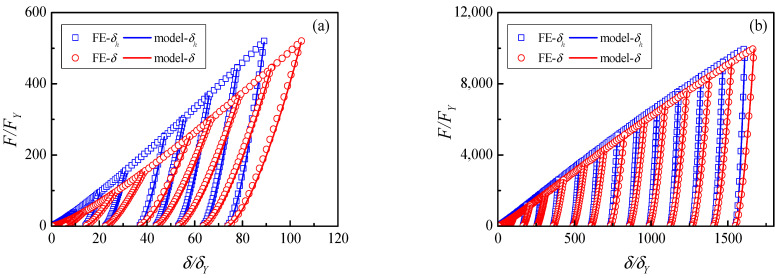
Comparisons of the unloading laws and FE results for different maximum indentations with the material of case (3): (**a**) small indentations, (**b**) large indentations.

For more contrast of the unloading process, many unloading simulations are performed from the small indentations to large indentations with the material of case (3) in Table 1. Figure 9 shows the indentations of total deformation and the half-space. The comparison of small deformations is plotted in Figure 9a, where the range of contact indentation $\delta_{m}/\delta_{Y}$ is from 0 to 100. The comparison of large indentations is plotted in Figure 9b, where the range of contact indentation $\delta_{m}/\delta_{Y}$ is from 0 to 1700. The unloading curves perform well regardless of small or large deformation.

### 3.6. Comparisons with Existing Unloading Models

**Figure 10:**
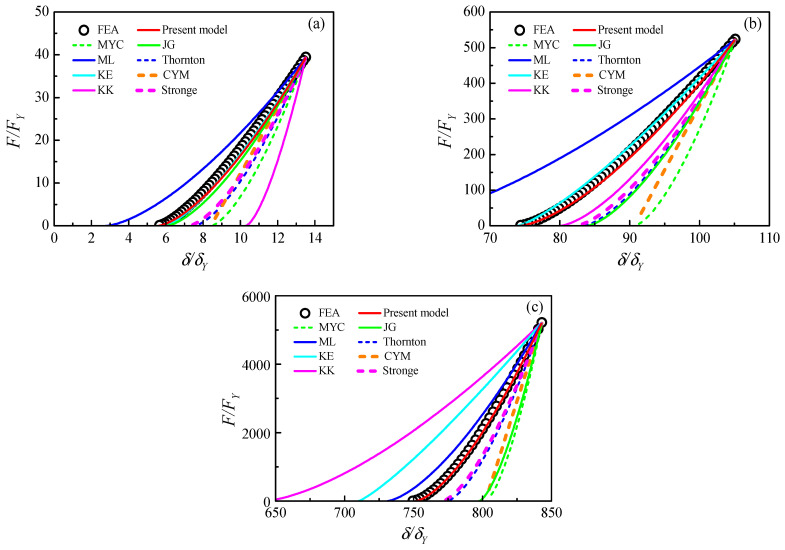
Comparisons of unloading models and the FE result with different maximum indentations for material case (3): (**a**) $13.5\delta_{Y},a/R^{*}=0.03$, (**b**) $105.1\delta_{Y},a/R^{*}=0.1$, (**c**) $843.1\delta_{Y},a/R^{*}=0.4$.

The unloading relationship of indentation and force is compared in Figure 10 with several existing contact models and FE results.

The unloading models are the Thornton model [28], KE model [24], KK model [34], Stronge model [27], JG model [51], MYC model [40], CYM model [42], ML model [32] and PLM model [23], respectively. Most of the unloading models are proposed only for the rigid and elastic–plastic contact problems, and the effective elastic modulus $E^{*}$ can be employed for the elastic and elastic–plastic contact. All the unloading models are calculated by the maximum indentation, force and contact radius from the FE result.

For the relatively small indentation in Figure 10a, the present model, KE model and JG model coincide well with the FE result. For the moderate indentation in Figure 10b, the proposed model and the KE model are perfectly coincident with the FE result; most of the unloading models predict a larger residual indentation than the FE result. For the large indentation in Figure 10c, only the proposed model shows good coincidence with the FE result; large differences can be observed for other unloading models during the unloading process. It illustrates the present unloading model can accurately predict the unloading behavior.

## 4. Applications

### 4.1. Contact Model

A complete contact model includes loading and unloading phases. Combining the present unloading law with the existing contact loading model, cyclical behavior including loading and unloading can be predicted. Weng et al. [23] investigated the deformation characteristics between an elastic–plastic half-space and an elastic sphere based on the FE results and provided a piecewise linear contact model (PLM) based on the linear characteristics. The PLM model can provide the contact force, total indentation, sphere indentation and half-space indentation during the loading process; therefore, the present unloading model can be employed with the PLM loading model.

**Figure 11:**
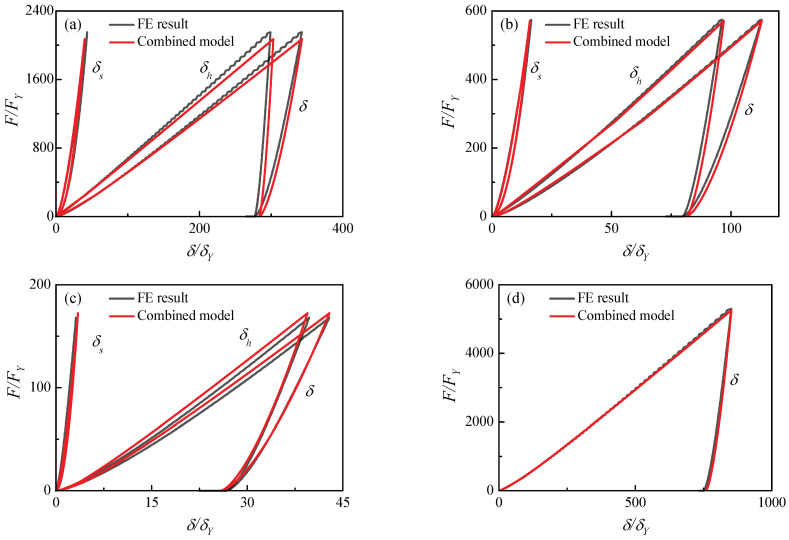
Comparison of present combined contact model and FE results: (**a**) material case (2), (**b**) material case (3), (**c**) material case (4), (**d**) material case (5).

Figure 11 shows the comparisons of the combined model and the FE results with the materials in Table 1. A slight deviation is found in Figure 11a,c and the combined model solves a lower contact force than the FE results. Great coincidences can be observed in Figure 11b,d. In Figure 11d for contact case (5), the elastic modulus of the hemisphere is large enough and can be considered to be rigid. Therefore, the deformation can be ignored. The comparisons illustrate that the combined contact model can accurately predict the evolution of contact indentation and force during the unloading process.

### 4.2. Impact Simulations

The unloading model combined with the PLM model [23] can be employed for low-velocity impact to examine the prediction accuracy. The impact simulation is performed by LS-DYNA R7.1.1 software. Five materials with low-yield stress are selected as the elastic–plastic half-space and three harder materials are selected as the elastic indenter. Four impact cases were simulated to compare with the combined contact model. More details and material combinations can be found in Table 7 in the literature [23].

**Figure 12:**
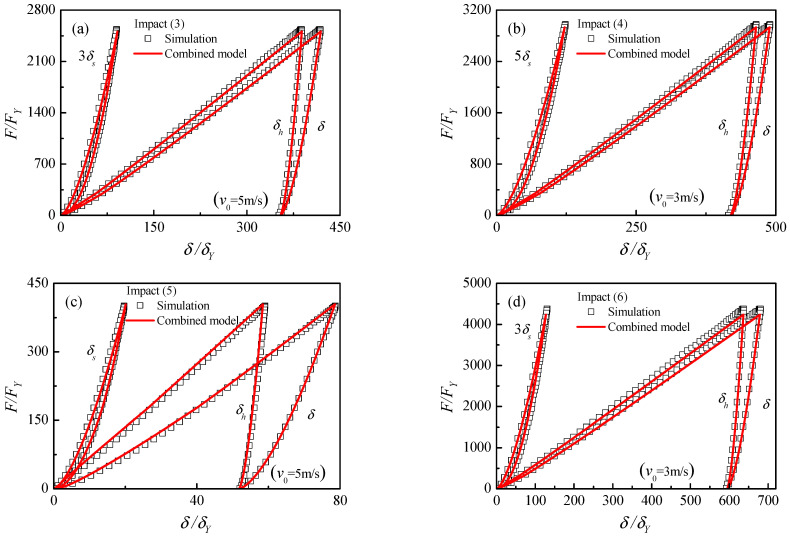
Comparison of present combined contact model and impact simulations: (**a**) $v_{0}=5$ m/s, impact (3), (**b**) $v_{0}=3$ m/s, impact (4), (**c**) $v_{0}=5$ m/s, impact (5), (**d**) $v_{0}=3$ m/s, impact (6).

Figure 12 presents the simulation and calculation curves of impact force versus δ, $\delta_{s}$ and $\delta_{h}$ for the impact cases (3–6) with $v_{0}=$3 and 5 m/s. To compare the results clearly, the indentations of the elastic sphere $\delta_{s}$ are magnified several times. The comparisons show perfect coincidence between the prediction and simulation results. It illustrates that the combined model can be employed for the low-velocity impact of elastic–perfectly plastic materials.

### 4.3. Nanoindentation Test

The combined contact model including the present unloading law is applied to the static indentation experiments of a hard sphere indenting into elastic–plastic material using spherical instrumented indentation tests [14]. The indented materials were deposited by an additive manufacturing process with pure titanium and pure niobium powders, and four different Ti-xNb alloys with the following Nb wt%: 5%, 10%, 40% and 70% were employed to validate the present contact model [14]. According to the strain–stress curves of each alloy obtained from the tensile test, the mechanical properties show low strain hardening and can be considered as elastic–perfectly plastic materials. The maximum tensile strength is more suitable for characterizing the plastic properties, and the Poisson’s ratio of the four Ti-xNb alloys is selected as 0.3. Table 3 shows the mechanical properties of the four Ti-xNb alloys obtained from the tensile tests. The indenter used in the nanoindentation is almost spherical with a radius of 0.25 mm made of tungsten carbide. The Young’s modulus and the Poisson’s ratio of the indenter are equal to 600 GPa and 0.23, respectively.

**Figure 13:**
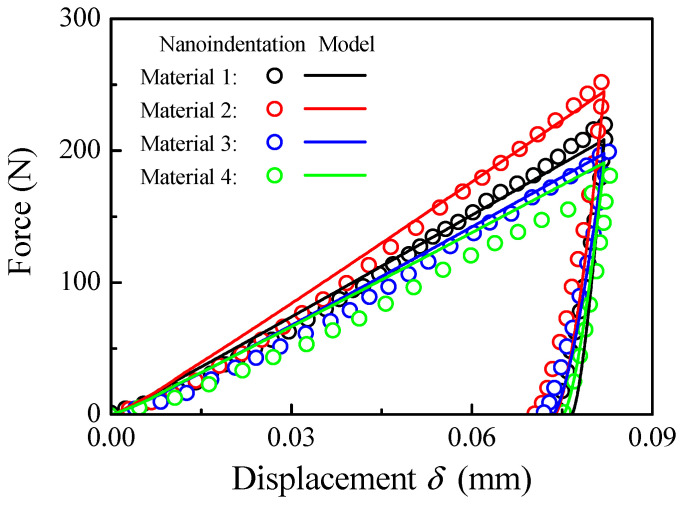
Comparisons of the indentation test results and present combined models.

Figure 13 shows the comparisons of the indentation test results and the present combined models. In the loading process, good coincidences can be observed for Materials 1–3 and a small deviation is observed for Material 4. In the unloading process, the permanent deformation predicted by the combined model is slightly larger compared with the indentation test results. The deviations can be attributed to the additive manufacturing process, as additive manufactured materials are heavily affected by residual stresses, which cannot be accounted for in the present model. Therefore, the predictions of the combined model can be applied to the nanoindentation test.

## 5. Conclusions

A theoretical unloading model of an elastic–plastic half-space contacted by an elastic sphere is presented to obtain high-accuracy predictions of the indentation–force relationship of unloading. The model is derived based on Hertzian elastic loading contact theory and depends on the elastic deformation of the sphere indenter. The proposed unloading model has been verified by static FE simulations, model comparisons, low-velocity impact simulations and a nanoindentation test. The result illustrates the proposed unloading model can accurately predict the unloading behavior. The main conclusions are as follows:

- A new unloading contact model of an elastic–perfectly plastic half-space indented by an elastic spherical sphere is presented analytically. The model can accurately predict the residual indentation and residual curvature radius after fully unloading.
- The assumptions and the unloading model have been demonstrated by numerical simulations. The proposed unloading can cover a wide range of indentations and material properties and is compared with existing unloading models.
- Combining the present unloading law with the existing contact loading model, cyclical behavior including loading and unloading can be predicted. The combined model has been verified by static FE simulations, low-velocity impact simulations and nanoindentation tests.

## Figures and Tables

**Table 1:** Material property of the sphere and half-space.

Case	$E_{h}$ (GPa)	$E_{s}$ (GPa)	$\sigma_{Y}$ (MPa)	${E_{h}^{*}/E}_{s}^{*}$
1	210	52	50	4
2	210	104	120	2
3	210	208	345	1
4	52.5	208	550	0.25
5	210	210,000	345	0.001

**Table 2:** The error of residual curvature radius.

Case	$R_{res}$-Fitting	$R_{res}$-Equation (25)	Error (%)
1	36.68	36.57	−0.30
2	37.53	37.26	−0.72
3	39.28	39.20	−0.20
4	41.12	40.55	−1.39
5	36.37	36.28	−0.25

**Table 3:** Mechanical properties of the Ti-xNb alloys determined from tensile test [14].

Materials	Elastic Modulus (GPa)	$\sigma_{m}$ (MPa)
1	100 ± 12	510 ± 23
2	77 ± 10	630 ± 22
3	58 ± 8	513 ± 19
4	70 ± 7	480 ± 4

## Data Availability

The original contributions presented in the study are included in the article, further inquiries can be directed to the corresponding author.
